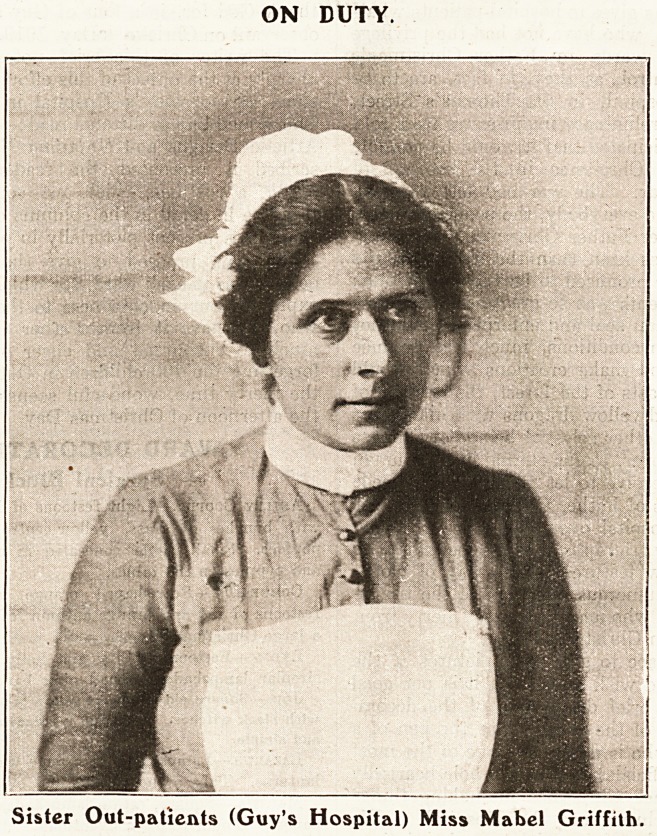# The Poor Children's Palace of Desire

**Published:** 1920-01-03

**Authors:** 


### IN TOUCH WITH THE OUTSIDE WORLD.

### The Poor Children's Palace of Desire.

Talking recently to a really great man about the measure of work done by men and women in various professions and occupations, and the added burden cast upon the real workers of the world by after-war conditions, the great man laughingly referred to the wonderful amount of work credited by the average person to learned professors holding high-sounding offices, whose work in fact, when measured and compared with that done by those who hold important office in voluntary hospitals with a medical school, is relatively insignificant. His view was, and it is the correct one, that to a man who really is a man, full-grown mentally and physically with a soul in his body and a working brain in his head, there is no place on earth where occupation can be so strenuous, so interesting, so uplifting, and so wonderful for its fruitfulness in results for good to the majority of his fellows with whom he is brought in contact as it can in a voluntary hospital of the best. A life's experience demonstrates that once a man or woman becomes absorbed in hospital work in the highest and best meaning of the word, for them at least there is no place like a voluntary hospital.

### How to Visit a Great Hospital.

In another page we give an outline of the artistic side to be seen at Christmas in a hospital, where the life, practice and results demonstrate the force and truth of every word that we have just written. It has so many sides as well as so many departments of care and cure, that a great hospital to-day cannot fail to prove an awakening and wonder-yielding machine for the independent enquirer and student of mankind. It is the fact that such a person can visit a great hospital many times, seeing something different or new on each occasion, and continue the practice, and yet end by not having seen possibly the most interesting and life-absorbing branch of a portion of the work, which may be seldom visited by, and little known to, any but the authorities and the workers who are responsible for the transaction of the business in such a department. On Christmas Day, for instance, at Guy's Hospital everywhere was evidenced life in its most active and self-denying form. Hundreds and hundreds of people of both sexes and all ages might be found in every portion of the hospital, giving of themselves and their time to the conscious and grateful occupation of ministering to the sick, the injured, the blind, the children, everybody who from no fault of his own is an inmate of the great sick-house, on the Day of the Nativity. Could a more fitting house of assembly be chosen for Christmas Day?

**THE ORIGINATOR OF THE LANDON TEAS.**

**Dr. Landon of Guy's Hospital.**

The Children's Very Own Department.

And yet it is true that there was one department devoted on that day wholly to the children, which visitors seldom or never saw, because the space available was strictly limited. In view of the number of little ones to be provided for, and the fact that things were to be made splendidly enjoyable and memorable for them, all other considerations were sacrificed. Here for five or more years Sister Out-patients has worked in charge of the out-patients department. She is a well-known force in the hospital world, is a woman of considerable parts and a strict disciplinarian, but one of the kindest and best beloved of women to be met with in the hospital field. Her organising powers are remarkable; how remarkable will become apparent to the administrator who learns that each year, with the aid of the Christmas Tea Committee, she organises a festival for Christmas Day for 700 children. These 700 children come from some of the poorest homes in the crowded districts of Bermondsey and Southwark. The day is eagerly looked forward to throughout these districts, where parents and children have grateful hearts and most lively anticipations every year as Christmas comes round. The children are brought to the hospital by the parents about 1.30, and at 4.30 the parents return to collect their offspring, bearing them away in triumph, for every child is laden with toys and other gifts and exuberant in smiles, resting on eager content and a merry heart.

A Word to Every Hospital Chairman.

A story and a lesson for every hospital chairman, committeeman, and governor attaches to the history of the establishment of the Children's Day at Guy's. We hope its recital may lead many hospital authorities to establish their Children's Day for the poor children who have sometimes been inmates of their institution, and who may reside in or near its vicinity. Twenty-two years ago, at Christmas-time, a fourth-year student, Mr. Landon, who was a popular character in his day, saw the Boro' children from the slums contiguous to Guy's Hospital crowding round any spot from which they could get a glimpse of the festivities within or of the Christmas-trees and gaieties in the wards at Christmas. Mr. Landon had a tender heart, and in the last year of his studentship he became determined that these slum children, or at least those of them who had been patients at Guy's, should have a tree of their own. The observant in large cities must often have noticed, not particularly to-day, but throughout the years of their life, that at this season there are often small crowds or gatherings of children, sometimes accompanied by their elders, who frequent the better streets and squares and gaze with wonder and interest at the lighted and crowded tables to be seen through the windows, and watch the arrival of gaily-dressed guests. The Borough is not the only place, alas, where the view which touched the heart of the late Dr. Landon is to be seen in all its pathetic force and lesson! There is not a city or town or humbler community in Great Britain where a modern Landon, with eyes to see and a heart to feel, cannot find Christlike work to his hand, if he or she has the soul and the will to undertake it.

How Mr. Landon Began.

Well, Mr. Landon, being a man of action, enlisted the interest of his friends and the co-operation of the authorities at Guy's, who approved his plan. They organised two large Christmas-trees, with a bountiful supply of Christmas things dear to the heart of the child, including the best of good fare, and collected some 500 children at Christmas 1897. It was a glorious day, not only for the children, but for their friends who looked after them. Amongst those friends were twelve sturdy policemen, accompanied by a sergeant, to keep order and guard against any stampede or accident. Those policemen, once enlisted in this service, never fail to be present year by year, and it is one of the instructive lessons, which Londoners who have eyes to see may learn, that the relation between the policeman and the child is one of perfect confidence carried to the limit of tenderness in the handling of the children by the men, and of affectionate trust in the sportsmanlike instincts and fatherly kindness of the man on the part of the child. Mr. Landon's venture and his interest in it were continued until his death in 1912, and every year funds were raised by subscription amongst the governors, students, and nurses at Guy's. On his death it was agreed by the House Committee, on the recommendation of the Christmas Tea Committee, that for the future the fund for the continuance of this work should be known as the Landon Tea Fund.

Some Poor Children in Excelsis.

On Christmas Day, 1919, as in every previous year on Christmas Day since 1897, 700 poor children had the time of their lives in the out-patient department at Guy's Hospital, which was specially prepared for them and splendidly organised by Sister Out-patients (Sister Mabel Griffith). No Londoner who has a heart should fail to learn more about Guy's Hospital, and so win his or her way by service to the right to at least one invitation to the Landon Tea. Each should make a point of becoming a life-subscriber to the Landon Tea Fund. If they succeed they will spend a wonderful afternoon. They will find over 700 Borough children sitting down to a table, facing from both ends to a central stage, on which the niggers gather and sing choruses. The children are all as keen as mustard. They are adepts at popular songs, and in consequence it is impossible for the niggers to do more than choruses, for at the first hint of a song known to the children, they take it in charge and the niggers are left far behind. The twelve tall policemen and the sergeant already referred to keep order and guard against a stampede.

**In Touch with the Outside World**—(*continued*).

The same men come year by year, for they love it. Many nurses, from the Matron downwards, take an active interest in all the arrangements, and for these, if they do their duty by trying to make their voices carry against the noise, hoarseness is as certain as that night follows day. Yet it would be impossible to find any one of the whole of them who did not declare with all her heart that it was worth it. After tea and wonderful singing in its most attractive form, too, every little sticky child is passed through a room where Father Christmas finds several presents from a lighter Christmas tree, including a game, books, sweets, an orange, what you will. In the result each child comes out laden with good things and is shepherded by the big policemen into the road where its parents claim it.

A GREAT FORCE AT GUY'S.

These Landon teas at Guy's on Christmas Day are a great force. Why they have escaped the eagle eye of the Press for so many years is a problem every well-conducted newspaper throughout the Metropolis may probably find a useful answer to, if it exists, in 1920. By that time too there should be many "Landon Teas" for children in great cities, brought into organised existence on Christmas Day 1920. And here it may be well to state that in the first year, 1897, roughs were outside with barrows and other vehicles. They robbed the children of their presents, and made off before their nefarious practices were apprehended and steps could be taken to make them abortive. Who shall say what difference twenty-two years have made in the manners and honesty of the rougher population throughout the Metropolis? We should hope, and, from our experience with our warriors at the front and at home we should make bold to declare, that if more Landon Tea Funds are started, and any roughs anywhere were to try a repetition of the 1897 tactics towards the little slum children, on emerging from a few hours' pleasure and enjoyment at a Landon Christmas Tea, they would have a warm time. They might, indeed, require more hospital care, when they were done with by the parents and our warriors, than they would be likely to forget for the rest of their lives.

**ON DUTY.**

**Sister Out-patients (Guy's Hospital) Miss Mabel Griffith.**

THE GLORIOUS PRIVILEGES OF OUT-PATIENT SISTER.

We venture to express our envy of the glorious privileges attaching to the offices of out-patient workers, from the residents to the porters, and from the matron through all grades of nurses to the wardmaids, to everybody who in 1919 has given their time, their talents, themselves, to make joyous the seven days during which Christmas and its attendant festivities are held throughout Guy's Hospital, for the cheer, uplifting, and enjoyment of its suffering and sick inmates. Our thanks are due to all workers in Guy's Hospital, who for many consecutive years have never spared themselves any possible trouble to enable us to give an account of the actual doings, their fruitful goodness, and the magnificence of the exhibition which they provide, of the glories of personal service in the days of health, in the cause of the sick.

## Figures and Tables

**Figure f1:**
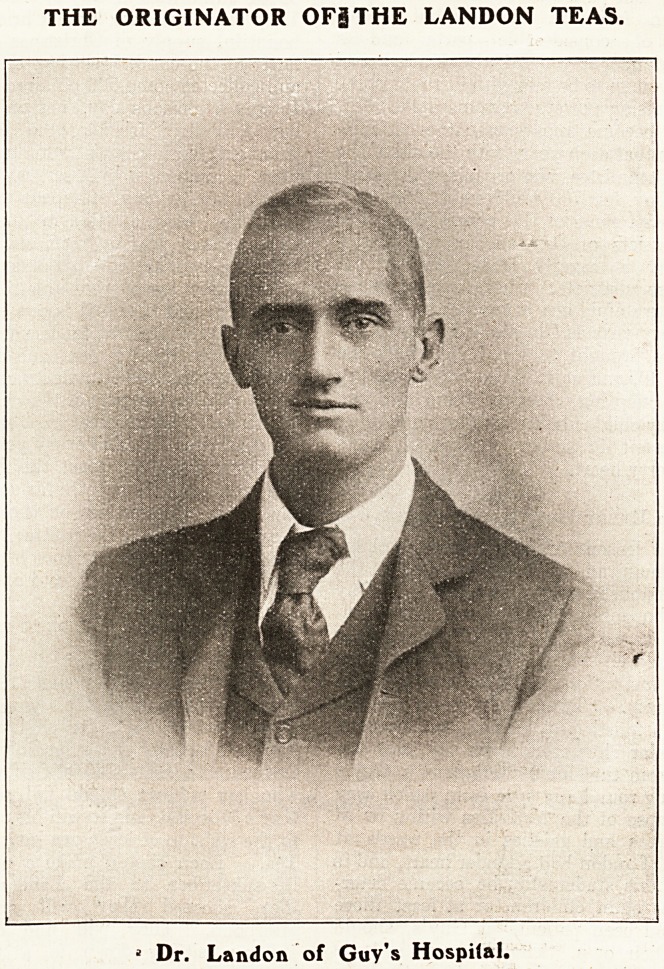


**Figure f2:**